# A Technique to Perform PCI and Stenting in an Anomalous RCA from the Left Sinus of Valsalva

**DOI:** 10.1155/2012/801423

**Published:** 2012-09-13

**Authors:** Dazhong Sun, Douglas Bogart

**Affiliations:** ^1^Kansas City Cardiology, 930 Carondelet Drive Suite 304, Kansas City, MO 64114, USA; ^2^Interventional Cardiology Section, Truman Medical Centers/University of Missouri-Kansas City, 2301 Holmes, Kansas City, MO 64108, USA

## Abstract

This describes a technique for the successful performance of percutaneous coronary intervention in patients whose right coronary artery arises from the left sinus of Valsalva and in whom the standard guiding catheters do not allow coaxial support.

## 1. Introduction

Percutaneous coronary intervention (PCI) can be difficult in patients who have a culprit lesion in a coronary artery that arises from the opposite sinus of Valsalva. These arteries can be difficult to visualize by coronary angiography and are even harder to cannulate to ensure guide stability for PCI.

We describe a technique that has been successful in patients whose right coronary artery arises from the left coronary sinus when the standard guiding catheters do not work.

## 2. Case Report

**Figure 1:**
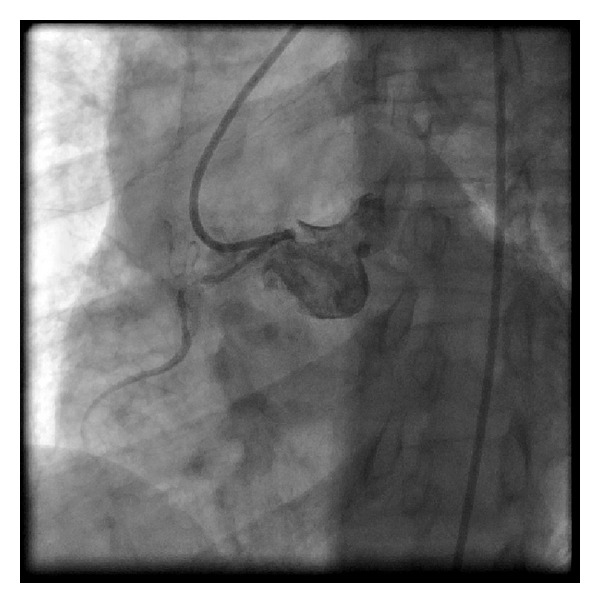
The difficulty in aligning catheters with the anomalous right coronary artery arising from the left sinus of Valsalva is shown here.

A 51-year-old African American female with a prior history of coronary artery disease presented to Truman Medical Center with chest pain and a non-ST segment elevation myocardial infarction. She had significant risk factors including type II diabetes mellitus, hypertension, dyslipidemia, and tobacco abuse. Her echocardiogram revealed mild inferior wall hypokinesis with normal overall left ventricular function. She underwent cardiac catheterization which showed only mild left coronary artery disease and an anomalous right coronary artery (RCA) from the left sinus of Valsalva (Figure 1). The RCA had a high-grade stenosis which was felt to be the culprit vessel. After attempts with multiple left guiding catheters (all 6 French (F)) (left Judkins JL 4, 5, and 6), left Amplatz [1–3], and the XB/series (3.0, 3.5, and 4.0) (all from Cordis Corporation, Miami, Florida), we were not able to get coaxial alignment for the PCI.

**Figure 2:**
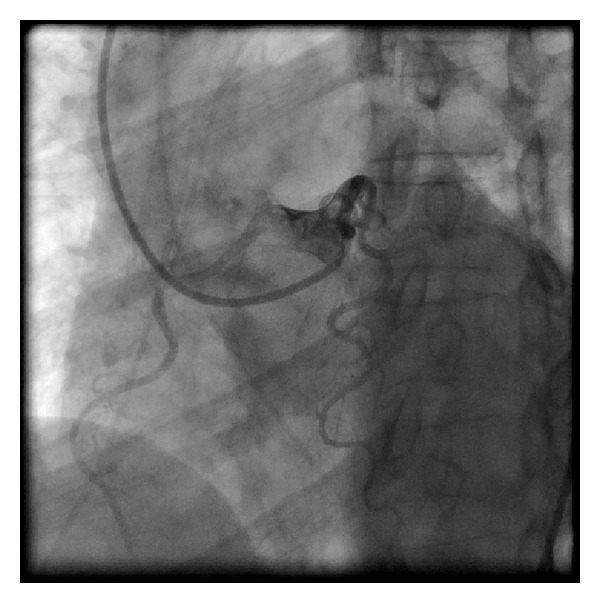
The 6 french left bypass catheter is aligned for wiring the anomalous right coronary artery.

We then used a 6 F left coronary bypass guide catheter which could be aligned for wiring of the RCA (Figure 2). We used a 180 cm Hi-Torque floppy wire and an over-the-wire 2.5 × 15 mm Voyager balloon (both from Abbott Vascular, Santa Clara, CA, USA).

**Figure 3:**
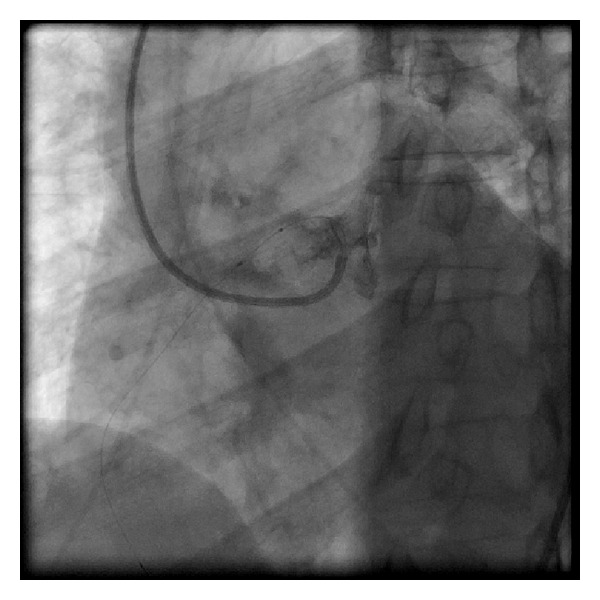
Wire cannulation is shown with the wire in the distal right coronary artery allowing for balloon passage and wire exchange.

**Figure 4:**
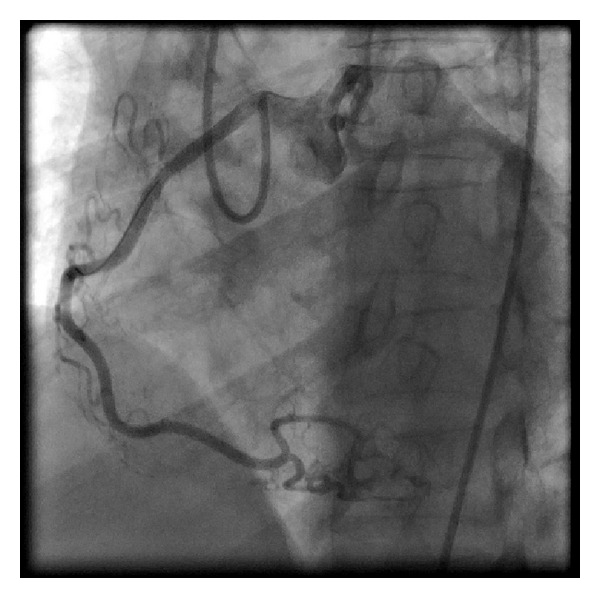
The completed result with the XB-3 guiding catheter well aligned demonstrating the final result.

We were able to place the wire in the distal RCA (Figure 3). We then exchanged for a 300 cm balanced middle weight (BMW) universal wire (Abbott Vascular, Santa Clara, CA, USA) which was placed through the balloon after removing the Hi-Torque floppy wire. We dilated the lesion and then removed the balloon. The left bypass catheter did not give adequate support to deliver the stent. We then exchanged the left bypass catheter over the BMW for an XB 3.0 guide (Cordis, Miami, FL, USA). With the BMW in place, we could engage the RCA and then completed the procedure (Figure 4). Two vision stents were deployed 3.0 × 28 mm and 3.5 × 15 mm (Abbott Vascular, Santa Clara, CA).

The angiographic result was good, and the patient was discharged the following day.

## 3. Discussion

It is frequently difficult to selectively cannulate the right coronary artery when it arises from the left sinus of Valsalva. The failure to adequately engage the anomalous vessel may lead to poor angiographic visualization and an erroneous diagnosis. Likewise, lack of guide support may lead to PCI failure.

The right coronary arises from the left sinus of Valsalva in 0.17% (136/126, 595) of patients undergoing coronary angiography [1]. Various catheters have been used to cannulate the anomalous vessel. These include the Amplatz-shaped catheters, the XB guide catheters, and the left Judkins catheters [2]. Very frequently multiple catheters are used with variable success. A new catheter curve (Leya catheter) has been reported to be of value for this difficult cannulation [3].

Our technique involves the use of a left bypass guiding catheter which is manipulated to align across from anomalous (RCA) (Figure 2) which allows for wire cannulation with a flexible wire and an over-the-wire balloon (Figure 3). We then pass the balloon into the distal RCA over the wire and exchange for a stiffer wire. If the stiffer wire allows for adequate support and visualization, we can complete the procedure with this guide. We have done this on two other patients. In this case, however, the support was tenuous and we used the XB guiding catheter which could be manipulated to engage the anomalous vessel (Figure 4). The procedure was then easily completed.

While this is somewhat of a labor intensive technique with the exchange of a guiding catheter over an angioplasty wire, it can convert a failed procedure into a successful one.
